# Sign language in the CT suite: a new approach in communicating between radiologists and radiographers

**DOI:** 10.1002/jmrs.173

**Published:** 2016-04-20

**Authors:** Edward H. Wang, Matthew J. Sampson

**Affiliations:** ^1^ Department of RadiologyRoyal Adelaide HospitalAdelaideSouth AustraliaAustralia; ^2^ Repat RadiologyRepatriation General HospitalDaw ParkSouth AustraliaAustralia; ^3^ Benson RadiologyNorth AdelaideSouth AustraliaAustralia

**Keywords:** Communication, non‐verbal communication, radiology, sign language, X‐ray computed tomography

## Abstract

When performing CT‐guided procedures or angiographic procedures, radiologists performing procedures need to communicate with radiographers at a workstation behind radiation shielding glass. As shielding renders verbal communication impossible, we have developed a set of standardised hand signals for use at our department to help us achieve clear and efficient communication between radiologists and radiographers while performing CT‐guided or angiographic procedures.

## Background

A good working relationship between radiologists and radiographers is important in order to ensure patient safety is maintained and that a high‐quality radiology service is provided.^1^ Good communication between radiologists and radiographers allows for a smoother workflow; poor communication, on the other hand, leaves a bad impression on patients.^2^ Studies have shown that most radiographers believe increasing the quality and quantity of inter‐professional communication improves patient care and job satisfaction, and that poor inter‐professional communication causes occupational stress.^3^

When performing CT‐guided procedures or angiographic procedures, radiologists performing procedures need to communicate with radiographers at a workstation behind radiation‐shielding glass. As shielding renders verbal communication impossible, we have developed a set of standardised hand signals for use at our department (Repat Radiology, Repatriation General Hospital, Adelaide, Australia) to help us achieve clear and efficient communication between radiologists and radiographers while performing CT‐guided or angiographic procedures. These hand signals are inspired by those used by scuba divers to communicate underwater without the use of voice communication equipment.^4^

To our knowledge, there is no existing literature discussing the topic of hand signals applied to the medical field. In our attempt to find relevant literature, extensive literature searches were performed on the database PubMed using different sets of search terms including ‘hand signals’, ‘hand signals radiology’, ‘hand signals medicine’, ‘sign language medicine’ and ‘sign language radiology’. No relevant literature was retrieved.

## Aim

The goal is to describe a set of hand signals developed and used at our department to assist communication between radiographers and radiologists during CT‐guided procedures or angiographic procedures. We find these signals useful in day‐to‐day practice and easy to learn. We hope other radiology departments or practices will also find these signals useful in assisting communication between radiologists and radiographers during procedures. Furthermore, these signals can potentially be adapted by other radiology practices or departments. Alternatively they could serve as inspiration for the development of more signals to further facilitate communication between radiographers and radiologists during procedures.

Our target audience includes radiographers, radiologists and radiology registrars.

## Hand Signals

Hand signals we have developed for use during CT‐guided and angiographic procedures include the following:

The ‘OK’ sign – universal – make a circle with thumb and index finger; extend other three fingers.

**Figure 1:**
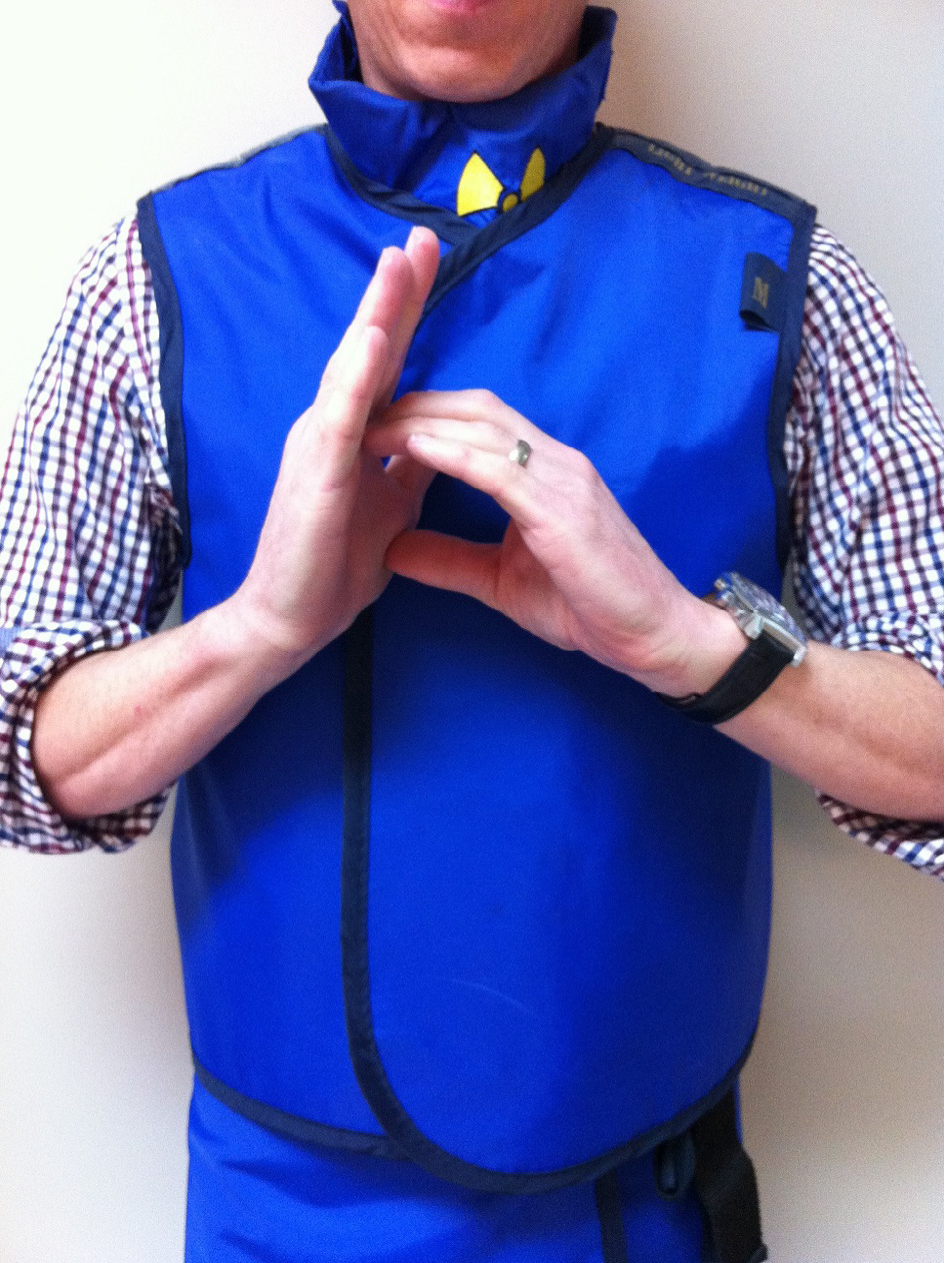
‘Code Blue!’

Code Blue/Emergency – used to call for help. Wave frantically, then make the letter ‘b’ with the palm of your right hand and the fingers of the left hand. Repeat until the message is understood (Fig. 1)

**Figure 2:**
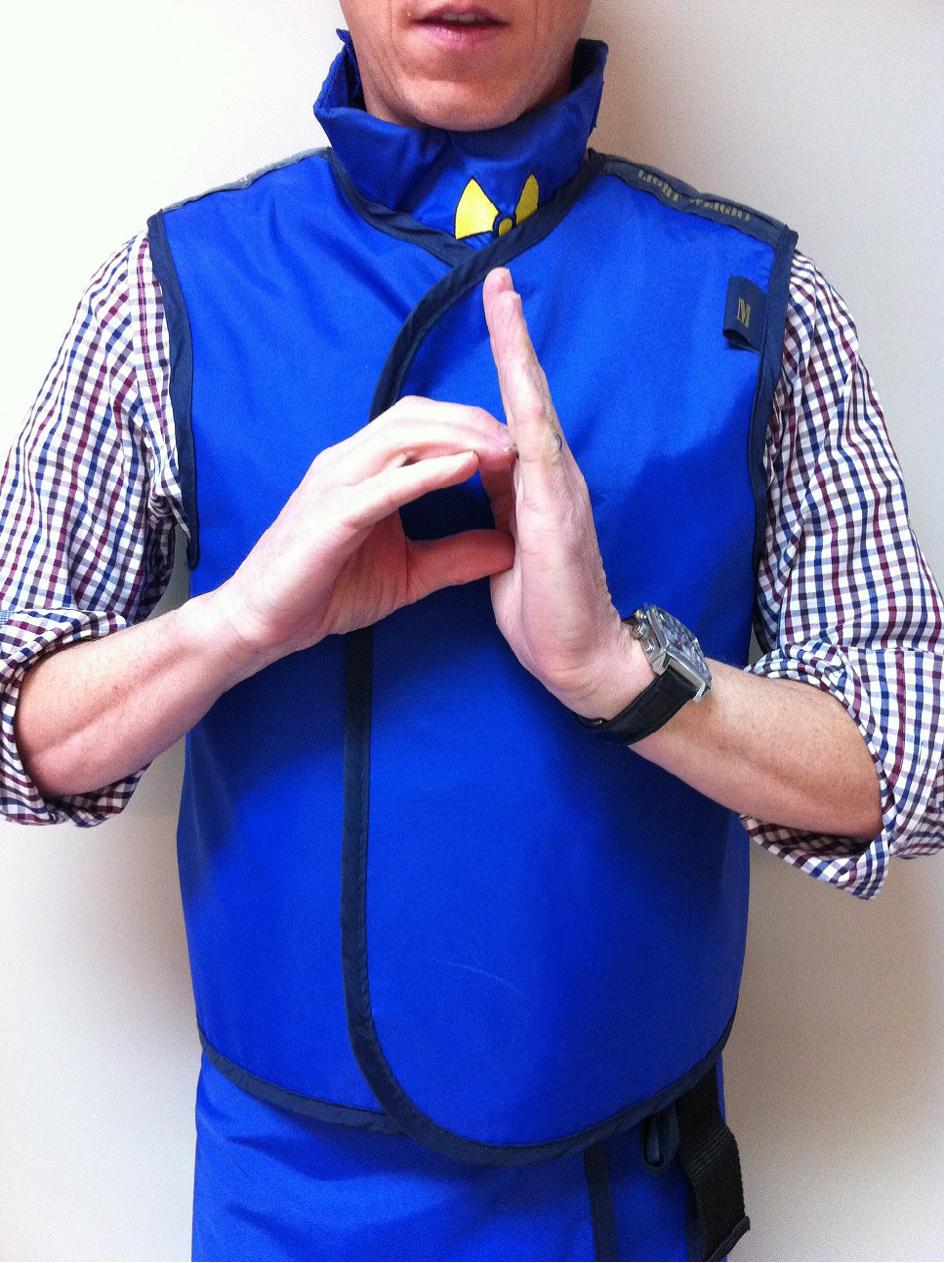
‘Measure distance.’

Distance – used to ask the radiographers to measure the distance to the target lesion and display it on the CT monitor. Make the letter ‘d’ with the palm of your left hand and the fingers of your right hand (Fig. 2)

**Figure 3:**
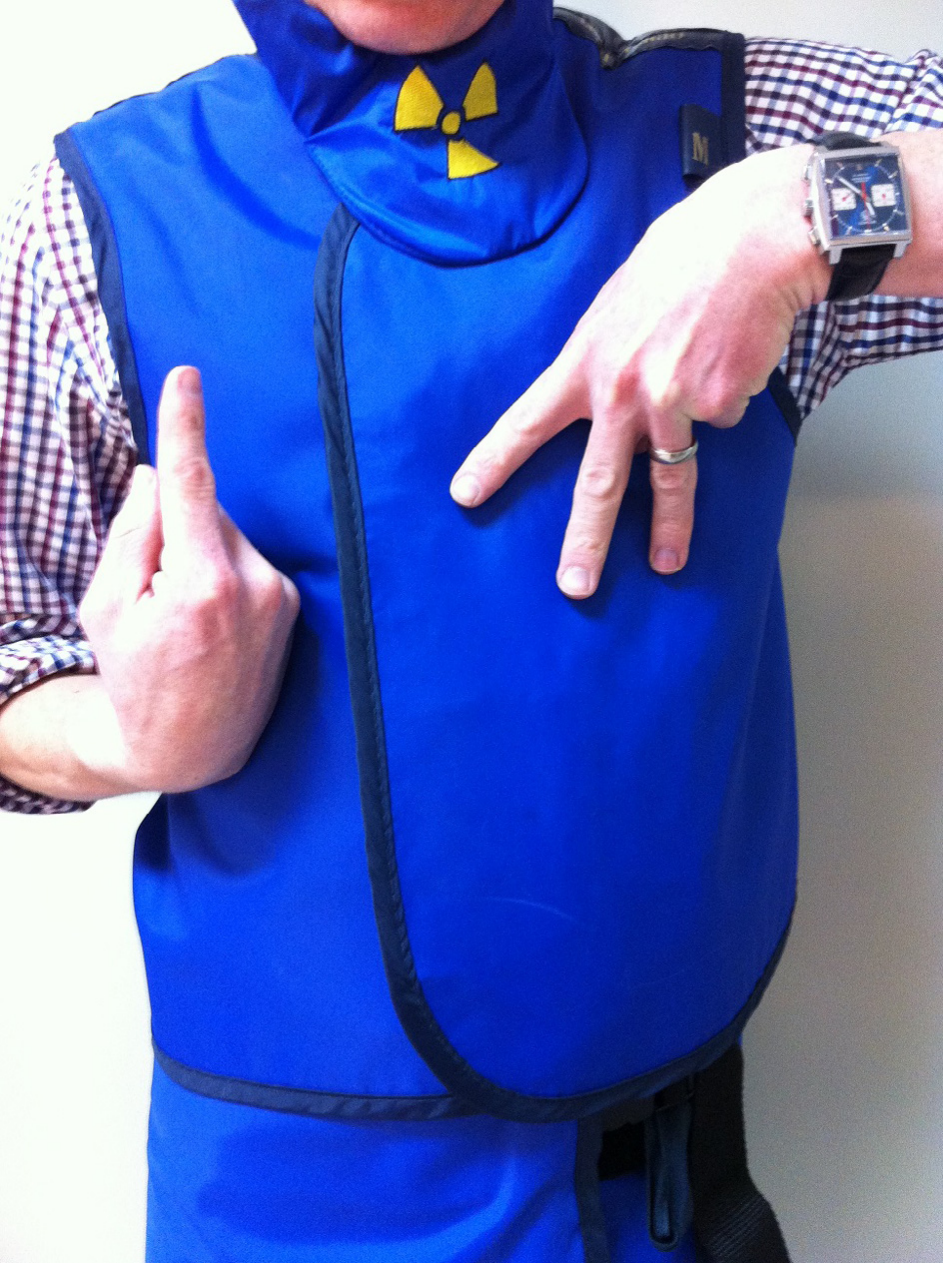
‘Zoom in.’

Magnification – for asking the radiographers to magnify the image displayed on the CT monitor. Extend the middle three fingers of one hand and point the hand downwards to form the shape of the letter M. With the other hand, point up to indicate to the radiographers to zoom in (Fig. 3), and point down to indicate to the radiographers to zoom out.

**Figure 4:**
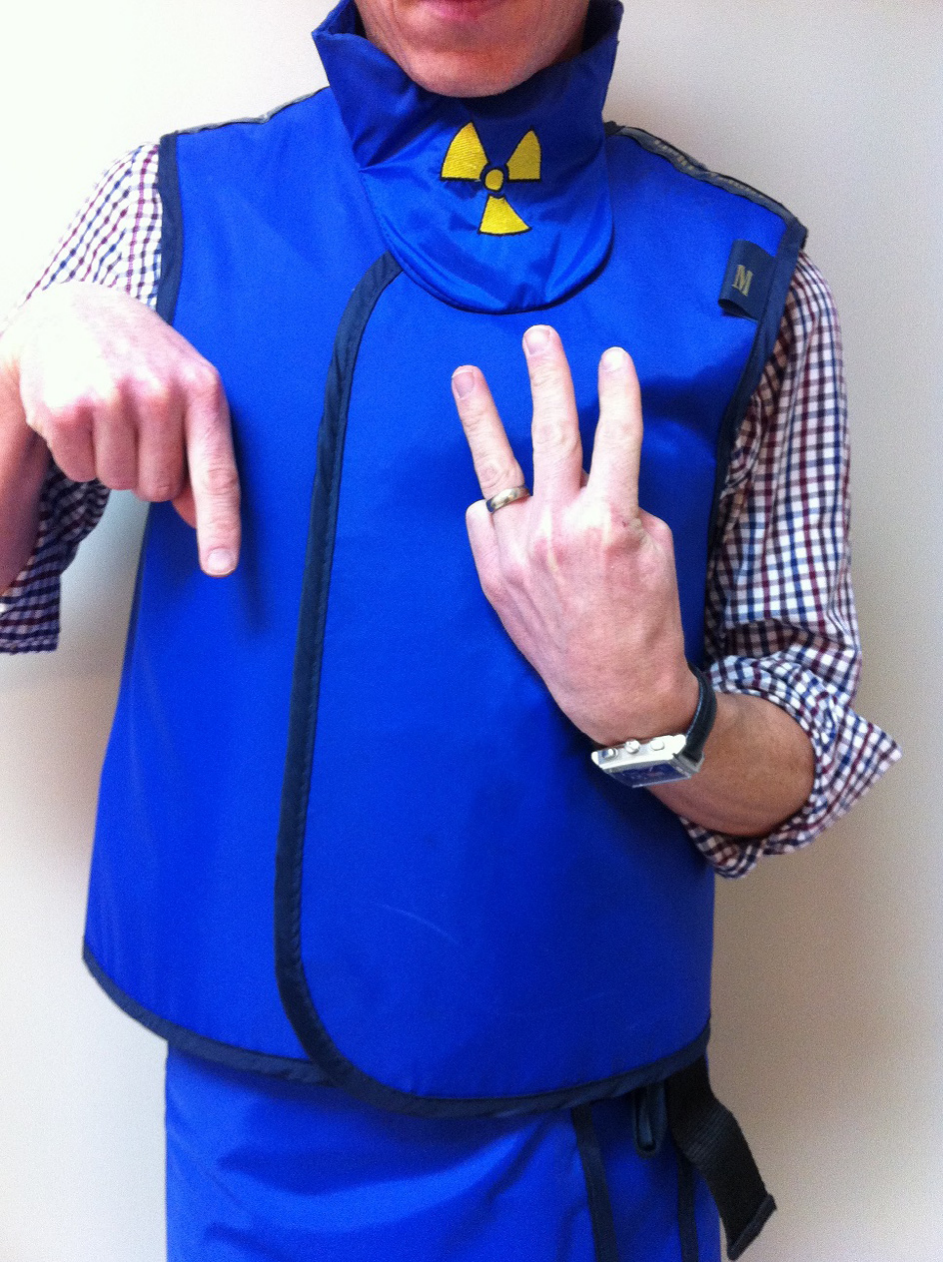
‘Narrow window width.’

Windowing – to alter the window width. Extend the middle three fingers and point this hand upwards to form the shape of the letter W. With the other hand, point up to signal the radiographers to widen the window width of the displayed image and point down to signal the radiographers to narrow the width (Fig. 4).

**Figure 5:**
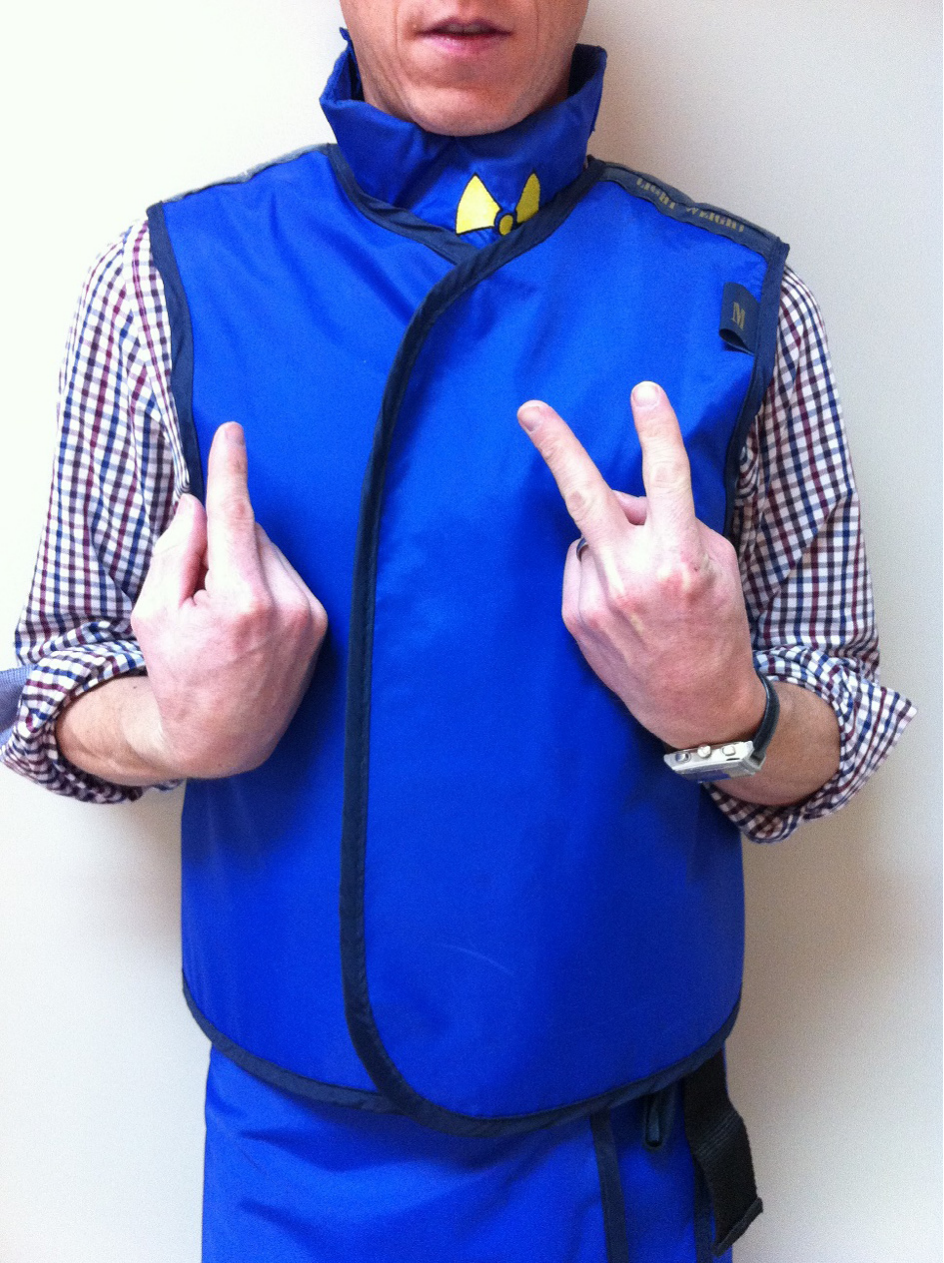
‘Increase voltage.’

Voltage (kV) – to alter the voltage during an angiographic procedure. Extend the middle and index finger and point the hand upward (to mimic ‘V’). With the other hand, point upwards to signal to increase the kV (Fig. 5) and point down to signal for the kV to be reduced.

**Figure 6:**
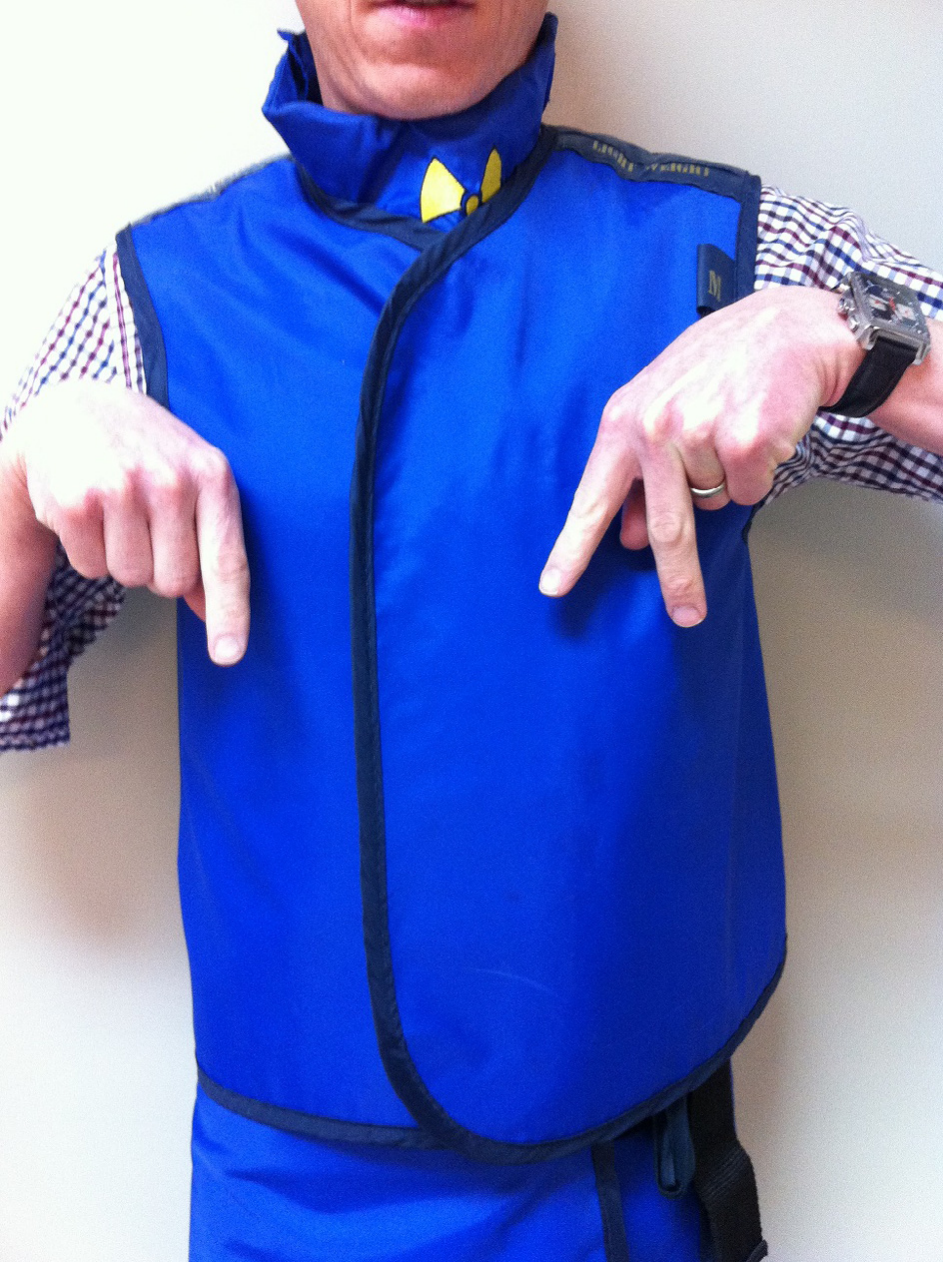
‘Decrease current.’

Current (mA) – to alter the current during an angiographic procedure. Extend the middle and index finger and point the hand downward (to mimic ‘A’). With the other hand, point upwards to signal that the mA delivered should be increased and point down to signal for the mA to be reduced (Fig. 6).

**Figure 7:**
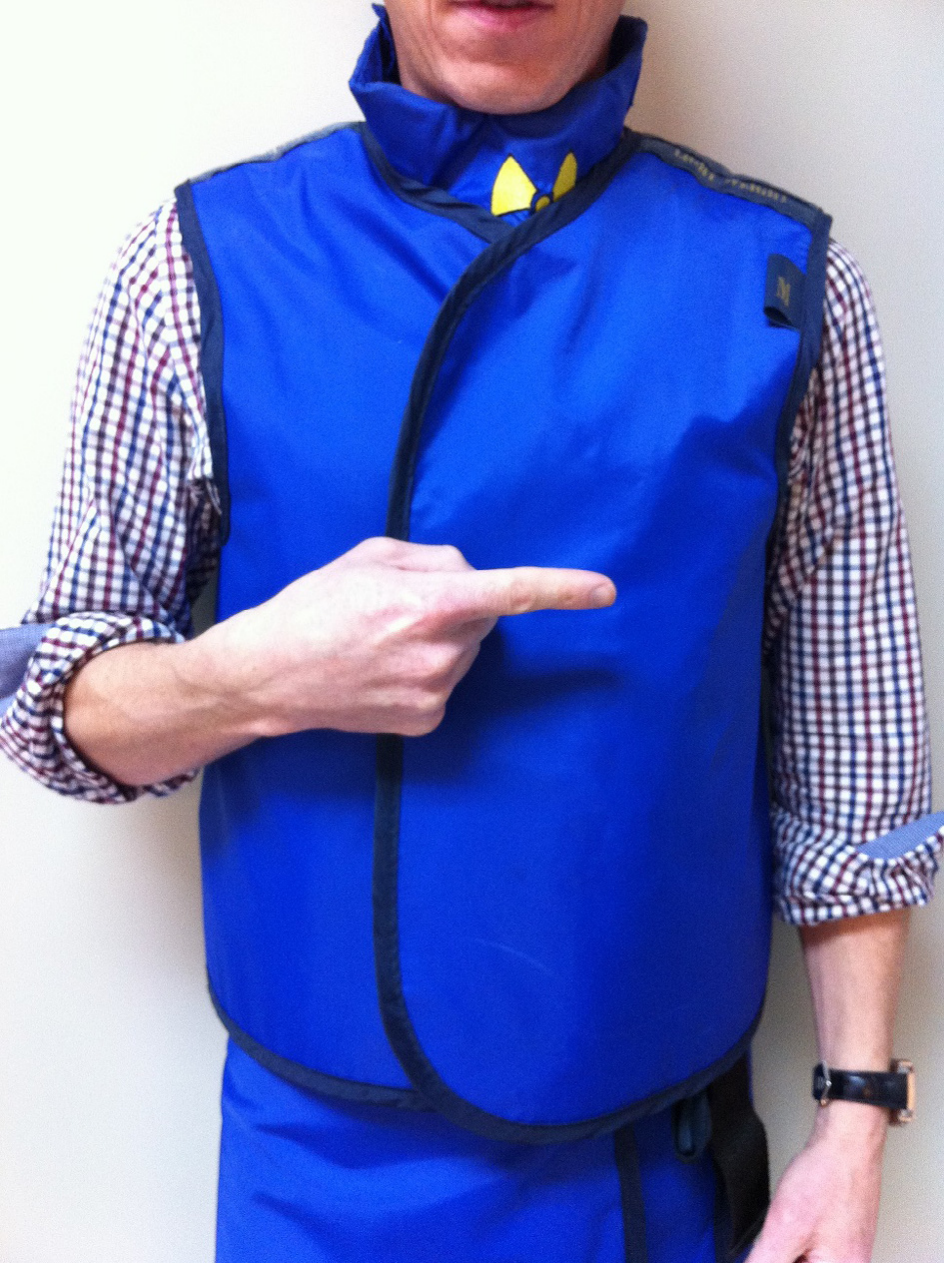
‘Table in.’

Table in – point in the direction of the CT gantry to signal for the table to be moved inwards. (Fig. 7)

Table out – point in the direction opposite to the CT gantry to signal for the table to be moved outwards.

## Conclusion

We have discussed a set of hand signals which are currently used in our department to facilitate communication between radiographers and radiologists while performing CT‐guided or angiographic procedures. These signals are useful and easy to learn. They can be adopted or modified by other institutions to help improve inter‐professional communication leading to better patient care.

## Conflict of Interest

The authors declare no conflict of interest.

## References

[1] Savoie B , Lexa F , Nagy P . Radiologist technologist communication. J Am Coll Radiol 2013; 10: 144–5. 23374693 10.1016/j.jacr.2012.10.014

[2] Kaplan D . The radiologist‐tech relationship: Why you should care. Diagnostic Imaging [Internet] 2015 [cited 2015 Jun 5]. Available from: http://www.diagnosticimaging.com/practice-management/radiologist-tech-relationship-why-you-should-care.

[3] Verhovsek E , Byington R , Deshkulkarni S . Perceptions of interprofessional communication: impact on patient care, occupational stress, and job satisfaction. Internet J Radiol 2010; 12.

[4] Common hand signals for scuba diving [Internet] 2005 [cited 2015 Jun 5]. Available from: http://www.neadc.org/CommonHandSignalsforScubaDiving.pdf.

